# Bibliography

**Published:** 1896-11

**Authors:** 


### Bibliography.

THE AMERICAN TEXT-BOOK OF PROSTHETIC DENTISTRY IN CONTRIBUTIONS BY EMINENT AUTHORITIES. Edited by Charles J. Essig, M.D., D.D.S., Professor of Mechanical Dentistry and Metallurgy, Department of Dentistry, University of Pennsylvania, Philadelphia. Illustrated with Nine Hundred and Eighty-three Engravings. Philadelphia and New York: Lea Brothers & Co., publishers, 1896.

The profession of dentistry in the United States has waited long and with some impatience for the appearance of a work worthy to be called a text-book upon the principles and practice of prosthetic dentistry, and at last it comes to us, not the labor of one man but of eight, with the guiding hand of an editor thoroughly trained, as must certainly be manifested throughout its pages.

The editor says in his preface, "There is contained in the body of the present volume the material which a consensus of opinion declares the best that dental prosthetic art has evolved." That this is true must be conceded by every careful and competent reader of the book.

The contributors have been selected for their recognized ability, and, apparently, for geographical distribution, as only three, including the editor, hail from Philadelphia, and the balance from the Middle States and far West. This must be recognized as a wise arrangement, and bars criticism in advance on the ground of local narrowness.

The work opens by a description of the "Mechanical Laboratory; its Equipment and Arrangement," by the editor, Dr. Charles J. Essig. This covers seventy-three pages, and is profusely illustrated, and will be invaluable to the beginner desirous of having a properly-appointed laboratory.

The succeeding chapter, on "Metals and Alloys used in Prosthetic Dentistry," by the editor, Dr. Essig, is, in the opinion of the writer, one of the most valuable contributions in the volume. While it does not include all of metallurgy, it does meet all the wants of the practitioner, and in the clearest manner explains processes so that the student should have a conception of the metals in daily use and the proper methods of treating them.

The next chapter, by Dr. C. L. Goddard, of San Francisco, seems to the reviewer to be very inappropriately named "Principles of Metal Work." The entire chapter is taken up with the preparation of appliances for regulating teeth. This does not detract in the least from its value, for it gives clear and satisfactory explanations of how these should be made, a very important matter not only to the student but to the advanced practitioner, who is often at a loss how to prepare apparatus for special cases. This chapter is also profusely illustrated in the main with artistic engravings. Throughout the book the careful reader will notice that in the illustrations, evidently prepared for the book, there is a close following of the natural forms of the teeth, these being, as a rule, very perfectly and beautifully drawn, and very satisfactory to the critical professional mind.

The chapter on "Moulding and Carving Porcelain Teeth," by Dr. Essig, is upon a subject of vital interest, and it is of special gratification that this has been prepared by one thoroughly conversant with it in its past history and practice. There is the ever-present danger of this being a lost art, as far as the study of dentistry is concerned. In the opinion of the writer, and one long entertained, this study should be made part of the curriculum of all colleges, for it certainly is the basis of all prosthetic work. It is observed with special gratification that the editor gives prominence to two skilled workers in this branch of prosthetic art, the late Professor Elias Wildman and W. R. Hall. The former failed to receive during his lifetime the full meed of praise due him, but for his efforts the manufacture of porcelain teeth would probably have remained as it was left by Stockton. His teeth have never been improved upon, and, indeed, it may be doubtful whether the perfection to which he attained has been continued by others. He prepared the first gum enamel worthy of consideration, and this, together with his formulæ for bodies, has never been improved upon.

The reader will find in this chapter very fully illustrated descriptions of the entire process of manufacturing teeth from the single plain to the gum tooth, and from the single gum to the block; the methods of carving teeth by hand; the different furnaces used; in fact, everything in relation to ceramic art as applied to the manufacture of teeth.

The article that follows on the "Preparation of the Mouth" is by Dr. H. H. Burchard. This chapter will probably meet with some criticism, not that it is out of the line of general practice, but that it will come in conflict with experience at many points. It impresses one with the difference that must ever exist between the laboratory and the office. An illustration of this is to be found in the author's idea of hyperæmia caused by the wearing of rubber plates. "Deposits of food-*débris* being permitted to remain upon the plate undergo fermentative and putrefactive decomposition, the products of which act as irritants. . . . Instances are seen where the wearing of a vulcanite plate, no matter how carefully finished or cleansed, is attended by hyperæmia of the underlying tissues." This the author ascribes "to lack of conductivity of the base." It is questionable whether this pathological condition is ever produced, where antiseptics are in daily use. The cause of this hyperæmia was demonstrated by Black to exist in the development of micro-organisms, and the old idea of mercurial action was exploded twenty-five years ago through the experimental work of Wildman, Buckingham, and Truman.

On page 276, on "Type of Denture," there seems to be more needed in relation to a peculiarly difficult class of mouths, where the author describes the "presence of tuberosities which cover the median line of the vault." In a paragraph in another article he attempts some explanation, but it is not very clear. A "horseshoe chamber" will not meet this difficulty, and, in the opinion of the writer, no impression was ever made by plaster from which a perfectly fitting plate could be made under this condition in the mouth. Every mouth of the kind requires hand manipulation with the plaster model, adding here and removing there. When this is properly done, a vacuum chamber will not be required.

"Taking Impressions of the Mouth," by the same author, is satisfactory and must be useful in suggestions.

The "Making of Models and their Preparation," by the same, is a very excellent and practical article, the illustrations, combined with the text, should render every operation clear to the student, showing the practical hand in every line.

"Dies, Counter-Dies, and Moulding," by the same writer, covers the entire ground of the work, and what has been said of the last chapter will be found true of this, and in this may also be included Chapter IX., on "Swaged Metallic Plates."

"The Bite or Occlusion" is the tenth chapter, and was given to Dr. Grant Molyneaux, and his work has been excellently done. He very wisely, in the reviewer's opinion, has incorporated Dr. W. G. A. Bonwill's method of articulation. For over twenty years this untiring inventor has labored to convince a careless dental world that the old methods of articulation were wrong in principle and worse in practice. It is simply amazing that his articulator has not even yet come into general use. Dr. Molyneaux has, it is thought, fairly given, in very full quotations from writings of Dr. Bonwill, his methods and theories, but it seems to the reviewer that, in all fairness, Dr. Bonwill should have had the privilege, in a work of this character, of presenting his own methods in his own way.

It will be impossible to follow up all the chapters, for the interested reader will understand that this review has not touched, except very lightly, on half of the seven hundred and fifty-one pages composing the book.

"Selecting and Fitting the Teeth" is a chapter of great practical value, and, did space permit, the rules formulated by Dr. Burchard for soldering would be repeated here. They should be placed in every college laboratory in letters large enough to be read daily by every student. No process in prosthetic dentistry is more difficult to understand than this, and comparatively few excel in it.

The chapter on "English Tube Teeth," by Dr. Essig, is one that has been greatly needed, for the ignorance regarding these valuable teeth on this side of the Atlantic is remarkable, but easily understood, as it has not been in the interest of manufacturers of porcelain teeth to make these aids to practice better known.

"Continuous Gum Dentures," by Dr. Ambler Tees, recalls to mind the valuable services rendered by the father in this direction, and the son has amply demonstrated his ability in this line of work. This was to be expected after the many years spent as demonstrator of "continuous gum," at the Dental Department of the University of Pennsylvania. To those at all familiar with working porcelain bodies and enamels these pages will be quite sufficient, but to those who have not been thus favored, the writer would advise practical instruction before attempting the insertion of this form of denture.

"Cast Dentures of Aluminum and Fusible Alloys," by Dr. C. L. Goddard, follows, and is a clear exposition of this part of the subject-matter.

"Vulcanized Rubber as a Base," by Dr. Essig, is a very interesting chapter and fully illustrated. It covers the entire subject, including the preparation of interdental splints, an important matter to the isolated practitioner.

"Celluloid and Zylonite" was given to Dr. W. W. Evans, whose rare ability in working these materials has long been recognized. The entire process is satisfactorily elucidated.

Dr. Alton Howard Thompson, of Kansas, discusses the "Temperaments," in Chapter XVII. This is a valuable contribution from the scientific and artistic side of the mechanical question, and the tables will, doubtless, often be referred to by those desirous of excelling, but it is expected the matter will be more highly appreciated in the next century than it will be in this by the average dentist. The standard of mental training has not, as yet, reached this, to the reviewer, very essential part of prosthetic dentistry.

In the chapter on the preparation of "Artificial Crowns," by Dr. Burchard, there will be found some erroneous teaching. "The pain following arsenical application is caused, in great part, by the pressure of the retaining filling-material." The action of arsenic is primarily to produce violent inflammation, and the pain is the result of this. If inflammation be present from prior pathological conditions, pain will be increased. Pressure has something to do with pain, but only in a limited degree. This, however, on the whole, is a valuable chapter, as is also that of the succeeding one on "Bridge-Work," by the same author.

"The Hygienic Relations and Care of Artificial Dentures," by

Dr. Essig, has not been a subject of special thought in works of this character, but is of very great importance, and it is a pleasure to allude to it as being part of the book.

The closing chapter on "Palatal Mechanism," by Dr. Rodrigues Ottolengui, is a fitting ending of this able production. The long and intimate relations existing between Dr. Kingsley and Dr. Ottolengui make this chapter of special value, as it covers the experience of two very able workers in this direction. It, in common with all the chapters, is properly illustrated. The fact that nine hundred and eighty-three illustrations are given in the book, and many of these of the most elaborate character, will furnish an idea of the expense attending its preparation; in fact, the publishers have spared nothing to make the work worthy the dental profession.

The writer of this review has gone over this book page by page, making this article of exceptional length. No other course could be adopted with a work of its importance. The errors, in his judgment, are but few, but, doubtless, many will take exceptions to many of the processes. There are occasionally blemishes, here and there, more in a lack of good taste than actual errors. It has been noticed that one writer frequently makes use of the French word *bête noire*, instead of using the plain but more effective English equivalent. Plain things need plain words to explain them, otherwise obscurity results and comprehension is rendered difficult.

The reviewer feels that the tendency of this book will be to raise the standard of mechanical dentistry, not merely in name, but in fact. It has too long been relegated to obscurity, and, in many instances, it has been regarded with contemptuous indifference. No one can fail to rise from the reading of the pages here presented without a feeling of increased respect for the basis of his profession, prosthetic dentistry, without which the scientific part would be as a sculptured figure without its pedestal.

The reviewer will be greatly disappointed if this book does not become the standard text-book in all colleges of the English-speaking world, for it is his decided opinion that no more thorough production will be found either in this country or in any country where dentistry is understood as a part of civilization.

The publishers deserve great credit for their liberality in placing this book upon the market. The work accomplished by this firm, in introducing the most valuable as well as the most expensive books on dentistry, should not be overlooked. Their "American System of Dentistry" was regarded, at the time of its issue, as a risk not likely to be repeated, and yet we understand a second edition of this is in preparation, as well as a companion to this just reviewed, a work on operative dentistry.

